# Towards the Optimization of a Photovoltaic/Membrane Distillation System for the Production of Pure Water

**DOI:** 10.3390/membranes14050110

**Published:** 2024-05-13

**Authors:** Dufei Fang, Damian M. Amiruddin, Imin Kao, Devinder Mahajan, Xuming Chen, Benjamin S. Hsiao

**Affiliations:** 1 Chemistry Department, Stony Brook University, Stony Brook, NY 11794-3400, USA; damian.amiruddin@stonybrook.edu; 2 Mechanical Engineering Department, Stony Brook University, Stony Brook, NY 11794-2300, USA; imin.kao@stonybrook.edu; 3 Materials Science and Chemical Engineering Department, Stony Brook University, Stony Brook, NY 11794-2275, USA; devinder.mahajan@stonybrook.edu; 4 SLB Brookshire Elastomer R&D Lab, 29501 Katy Freeway, Katy, TX 77494, USA; xchen51@cameron.slb.com

**Keywords:** pure water, photovoltaic, membrane distillation, electrolysis, hydrogen production

## Abstract

The production of pure water plays a pivotal role in enabling sustainable green hydrogen production through electrolysis. The current industrial approach for generating pure water relies on energy-intensive techniques such as reverse osmosis. This study unveils a straightforward method to produce pure water, employing real-world units derived from previously simulated and developed laboratory devices. This demonstrated system is cost-effective and boasts low energy consumption, utilizing membrane distillation (MD) driven by the waste heat harnessed from photovoltaic (PV) panels. In a previous study, modeling simulations were conducted to optimize the multi-layered MD system, serving as a blueprint for the construction of prototype devices with a suitable selection of materials, enabling the construction of field-testable units. The most efficient PV-MD device, featuring evaporation and condensation zones constructed from steel sheets and polytetrafluoroethylene (PTFE) membranes, is capable of yielding high-purity water with conductivity levels below 145 μS with high flux rates.

## 1. Introduction

The utilization of electrolysis for green hydrogen production presents novel prospects in the context of energy storage for dynamic and intermittent power generation, such as that driven by solar and wind resources. This arises from the fact that hydrogen, when employed as a fuel, facilitates the generation of electricity with water as the sole by-product. This process establishes hydrogen as an appealing choice for sustainable energy storage given its exceptional efficiency and adaptability, leading to the complete absence of carbon emissions. Furthermore, the adoption of hydrogen as a fuel holds promise in addressing the adverse impacts of climate change. However, within the realm of electrolysis, there is a prevalent preference for the use of pure water to ensure the optimal production of hydrogen. Put differently, the water source employed in the electrolysis process suitable for industrial operations should exhibit minimal contaminants and metal ions, characterized by high resistivity, even as high as 18 MΩ [1].

It is interesting to note that various approaches have been demonstrated to produce hydrogen directly from seawater. However, as the energy consumption and operational costs of these approaches are relatively high, none of them have been able to reach the commercialization stage for hydrogen production. For example, Guo demonstrated a seawater electrolyzer using Lewis acid-modified electrodes (Cr_2_O_3_–CoO_+_) that can hydrolyze seawater directly [2]. However, the long-term stability and reliability of this electrolyzer is still not proven, and it cannot be said with certainty that it can be used for large scale industrial applications. Xie reported a non-desalinated direct seawater electrolysis technology that can also generate hydrogen directly from seawater [3]. This technology includes porous membranes that can separate the electrolytes from the seawater. Specifically, the fluorine-rich membrane keeps liquid seawater out but allows pure water vapor to pass through. In this technology, a membrane is still required before electrolyzing the water. The driving force for the membrane separation is the salt concentration difference. However, as the concentration difference is relatively small, the low driving force somewhat limits the hydrogen production scale. So, seawater electrolysis technologies are still in the early research and development stages [4,5,6]. For commercial large-scale electrolysis to generate clean hydrogen, pure water is still an essential component for industrial applications.

There are currently many methods for industrial desalination of contaminated water, brackish water, and seawater. These methods are relatively high energy consumers and are not suitable for small-scale and/or off-grid operations. Common industrial methods for producing pure water primarily encompass the following steps: the source water initially undergoes filtration through activated carbon filters and/or UV disinfection treatments. The activated carbon treatment typically offers a cost-effective means of generating prefiltered water [7]. Following this step, the prefiltered water enters a reverse osmosis (RO) system to remove minute impurities, particularly metal ions. Reverse osmosis operates through the application of pressure, employing semi-permeable membranes for the purification of feed water [8]. It selectively prevents the passage of contaminants and ions larger than 0.5 nm in size (equivalent to the hydrated Na^+^ ion cluster). After RO filtration, the water can be subjected to an ion exchange step to eliminate any remaining ions. Activated carbon again plays a vital role here, enabling the physical adsorption of contaminants through chemical and physical interactions between the substrate surface, enriched with active functional groups and the contaminants [9]. However, it is noteworthy that complete pure water generation systems involving RO and/or ion exchange typically require substantial capital investments. Furthermore, conventional RO systems consume significant amounts of energy and utilize approximately three times as much water as they produce [10]. Excluding installation costs, pure water is valued between USD 0.15/m³ and USD 0.50/m³ [11]. Incorporating system installation and maintenance expenses would increase the overall cost significantly.

In this study, we demonstrate a facile technique to produce pure water, achieved through field-tested units based on a previously simulated and verified design of an integrated photovoltaic (PV)/membrane distillation (MD) system [12], characterized by economical capital and operational costs. MD represents a non-isothermal membrane separation process utilized for water purification. Within the MD system, a hydrophobic membrane divides the feed and permeate streams. The driving mechanism in MD relies on the partial pressure differential between these two streams, a consequence of the temperature variance. Previous demonstrations by Bodell and Weyl have established the suitability of this process for desalination and wastewater treatment [13,14]. Since then, various MD configurations were reported, with the field unit devices adopting a flat-top air gap membrane distillation (AGMD) system, leveraging the waste heat generated by PV panels. In the AGMD arrangement, a hydrophobic membrane serves as a barrier between the feed water and condensation channels. Direct contact with the hydrophobic membrane is maintained by the feed water, while an air gap, accompanied by a condensation plate, is present on the membrane’s opposite side. The hydrophobic membrane’s role is to permit vapor transmission while allowing the liquid feed water to pass through. The vapor travels through the membrane and air gap until it reaches the cooling plate on the opposing side, where it condenses into highly pure water.

**Figure 1:**
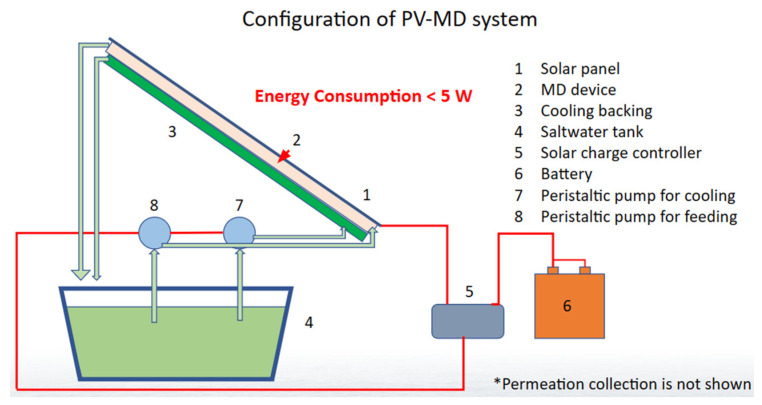
Initial configuration of PV-MD system design for field testing.

The process of MD hinges on an essential disparity in partial vapor pressure across the membrane, necessitating an external heat source. Notably, the Wang group has recently showcased the utilization of waste heat from solar panels as a viable energy reservoir for water purification through MD operation [15,16,17,18]. This approach is rooted in the fact that, despite the substantial energy absorption capacity of solar panels, conventional PV systems can only convert roughly 20% of incident solar energy into electricity, leaving the remainder to be dissipated as heat [19]. This surplus heat presents an opportune energy source, particularly when harnessed by a flat MD unit positioned beneath the PV panel. Such a configuration (Figure 1) can yield pure water, ideally suited for hydrogen production via electrolysis. For the current investigation, we employed a flat top PV-MD system for field testing to determine the efficiency of previously simulated and indoor tested devices. The multilayered MD unit’s design initially underwent computational simulation, with meticulous material selection to assess the unit’s operational efficacy. After small scale testing, it was scaled up to field testable units. 

## 2. Experimental

### 2.1. System Design Considerations

In our earlier study [12], a detailed description was given to describe the selection of separation materials—particularly the PTFE membrane—and the multiple layer configurations. In this work, we emphasize the engineering portion of the design considerations and the practical issues of the selected materials.

#### 2.1.1. Top Thermal Conducting Plate

In our design, we account for the heat emanating from the rear surface of the PV panel, which serves as the primary source of thermal energy for the entire MD system. In the subsequent layers of the system, the latent heat resulting from the condensation process within the final assembly plays a pivotal role in sustaining the required heat flux. The uppermost thermal-conductive plate also plays an important role in efficiently conducting and transmitting heat to the remainder of the system. This principle is extended to all other condensation plates within the device, necessitating proficient heat conduction and distribution capabilities.

#### 2.1.2. Feed Water (Evaporation) Channel

In the field-tested units, the feed water employed is a synthesized seawater solution achieved by dissolving an appropriate quantity of NaCl salt to attain a concentration of 35 g per liter. This solution is introduced into one end of the assembly layer at an exceptionally low flow rate, with the water temperature elevated through the thermal conduction from the uppermost plate of the assembly.

#### 2.1.3. Hydrophobic Porous Membrane

The pivotal component in the design of the PV-MD system is the hydrophobic membrane. This membrane must exhibit a very high level of hydrophobicity and porosity. Here, water undergoes evaporation at the membrane’s surface, and the resulting water vapor permeates through the porous structure. Despite the water vapor’s ability to pass through, since metal ions are comparatively larger, they are effectively barred from passage by the membrane. This selective permeation ensures that only pure water can traverse the membrane, ultimately accumulating on the condensation side.

#### 2.1.4. Air Gap

The vapor is generated through the porous membrane, and is propelled by the partial vapor pressure differential and then directed into the air gap to instigate the condensation phase on the designated condensation plate. Within this air gap, water vapor is present because of its transport through the membrane, and the humidity levels tend to approach saturation. As the vapor reaches the condensation plate, it undergoes condensation, giving rise to pure water. The latent heat released during this condensation process is harnessed in the underlying layer, serving as the heat source for subsequent stages.

#### 2.1.5. Condensation Plate

The condensation plate marks the terminus of the air gap where the water vapor undergoes condensation, releasing heat that is subsequently conveyed to the subsequent layers. The material chosen for this component must possess excellent thermal conductivity. Additionally, it is imperative to guarantee that the material is corrosion-resistant, thereby preventing any inadvertent ion contamination from compromising the purity of the condensed pure water.

#### 2.1.6. Cooling Channel

Positioned immediately following the final assembly layer’s condensation plate is the cooling water channel. This conduit accommodates the circulation of ambient-temperature water, effectively dissipating any lingering latent heat, thereby ensuring the establishment of a consistent temperature gradient throughout the multilayered assembly. The cooling water flow functions as the thermal reservoir, with its efficiency closely tied to the specific characteristics of the water flow rate. Previous simulation work has demonstrated that this layer significantly enhances the yield of pure water.

### 2.2. Materials Selection

The PV-MD system used in the field tests comprised five layers of 50 micron-thick 316L stainless steel condensation plates. The top casing plate was made of 0.9 mm-thick 316 stainless steel sheet, and the bottom plate was made of 0.75 mm-thick 316 stainless steel sheet, providing a total area of 1.5 m². These stainless steel plates offer corrosion resistance and mechanical strength. A water flow diversion cloth with epoxy coating, typically employed in purified water channels of spiral-wound reverse-osmosis cartridges, was used to define the feed water channel. The PTFE membrane, sourced from YOUKEFA Inc. in China, was approximately 50 microns thick with an average pore size of 0.45 microns. A 1.65 mm diamond-shaped polypropylene net spacer with a 1.0 mm thickness was placed in the air gap. Stainless steel plates were precoated with Goop gel, and PE Supreme Silicon and G/flex 605 were used for side sealing. The water-cooling plate was adhered to the stainless steel bottom plate with Sika 209D primer and Sikaflex 1a polyurethane sealant.

### 2.3. Assembly of the PV-MD System

**Figure 2:**
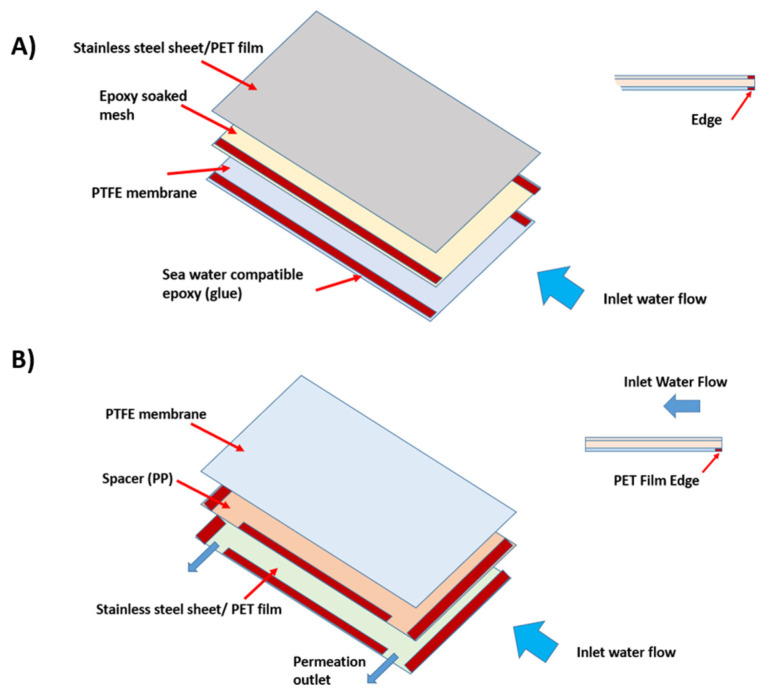
Assembly of MD system: (**A**) evaporation region, consisting of a sheet of epoxy coated fine mesh (or water diversion cloth) as spacer, and (**B**) condensation region, consisting of a polypropylene (PP) mesh as spacer.

The comprehensive schematic representation of the multilayered MD system is illustrated in Figure 2. (note: consider changing the location of the inlet water flow so as to better illustrate the entry points of the water. The current (A) and (B) makes it look as if the entry points are the same). The system’s operation is initiated as heat is absorbed by the uppermost thermal-conductive plate, which then conducts this thermal energy downward through the assembly layers. Each assembly layer can be conceptually divided into two distinct regions: the evaporation region and the condensation region. Positioned at the base of the ultimate assembly layer is a water-cooling channel designed to employ the same feed water as the cooling source. The feed water circulates through both the assembly layer(s) and the water-cooling channel, completing the system’s thermal cycle.

As the feed water enters the evaporation area, the difference in partial pressure facilitates the generation of pure water vapor from the feed water. This pressure difference is contingent upon the temperature contrast between the evaporation and condensation sections. Our simulation findings consistently revealed a similar partial pressure difference across the membrane in various assembly layers [12]. The resulting vapor transverses the membrane and subsequently condenses within the dedicated condensation channel of the assembly. Water that has undergone the evaporation phase can be recirculated back to the feed water source, effectively reducing the total demand for feed water. It is worth noting that when seawater is used as the source, the reintroduction of brine into the initial feedwater can lead to an increase in salinity, an aspect that necessitates careful consideration.

### 2.4. Assembly of Field Test Unit

The experimental PV-MD apparatus employed in this study is comprised of MD channels, peristaltic pumps, various flow conduits for water, and containers designated for feed water and the collection of pure water. The initial configuration (Design 1) is illustrated in Figure 1. This system contains a solar panel, MD system, cooling support structure, a saline water reservoir, a solar charge controller, a storage battery which is charged by PV panel through a smart controller, and a pair of peristaltic pumps. This assembly proved to be a suitable arrangement for preliminary assessments, showcasing the feasibility of outdoor testing. The peristaltic pumps were responsible for circulating both feed water and cooling water through the system, with their power supplied exclusively by the battery that is charged by solar panel. After this initial configuration, the system underwent two additional iterations, each manifesting as a progressive enhancement over its predecessor, with higher efficiency in producing fresh water.

**Figure 3:**
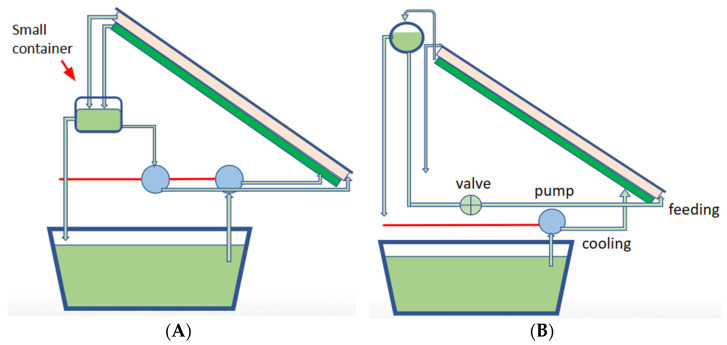
(**A**) Second field testing unit (Design 2) with small container of pre-heated feed water to utilize extra heat (left). (**B**) Third field testing unit (Design 3) with gravity-fed pre-heated water reservoir for the self-flow of feed water with a valve control (right).

The improved system (Design 2) is illustrated in Figure 3, where a notable modification was made. This configuration incorporated a compact reservoir situated above the peristaltic pumps and below the PV-MD system. Within this system, heat played a pivotal role in generating the requisite partial pressure for pure water production. The incorporation of this diminutive reservoir offered the opportunity to harness the waste heat from the cooling mechanism, thus elevating the temperature of the feed water. This augmented temperature was instrumental in intensifying the partial pressure differential and holds promise for enhancing the rate of feed water evaporation.

Although the Design 2 system exhibited better performance compared to Design 1, there remained further room for refinement. The third iteration of the system (Design 3), engineered for field testing, is depicted in Figure 3. In this configuration, a key enhancement involved the elimination of one peristaltic pump, resulting in a reduction of energy consumption drawn from the solar panel. Additionally, the relocation of the reservoir to a higher position within the system promoted a self-flow mechanism due to potential energy, eliminating the need for the second peristaltic pump while enabling the introduction of an adjustable valve to regulate the feed water flow rate. Notably, this system retained the same thermal management principles as the second design but realized energy savings from the solar panel by removing a pump. Another important factor to address here is that there is no significant change in the system efficiency between the two designs. However, although the system efficiency is similar, the amount of energy consumed decreases from Design 1 to Design 3. Furthermore, this system design exhibited improved performance, which will be discussed in greater detail subsequently.

## 3. Results

### 3.1. Lab Testing of Five-Layer PV-MD System

Preceding the assembly of the field-testing units, the construction and integrity of the PV-MD device were carefully investigated, with a major focus on detecting leaks or other engineering anomalies. The five-layer PV-MD apparatus featured middle condensation plates crafted from 316L stainless steel, each measuring 50 microns in thickness. The uppermost plate was constructed from 316 stainless steel, with a thickness of 0.9 mm, while the lower plate was 0.75 mm thickness of 316 stainless steel. To achieve hermetic sealing of the device, the stainless steel sheets underwent a coating process with Goop gel. The lateral sealing procedure entailed the utilization of PE Supreme silicone and G/flex 605 to securely seal the layers, thereby ensuring the prevention of any water leakage. A series of experiments with assessments were conducted over several days, simulating the complete temperature cycle from early morning to late afternoon.

**Figure 4:**
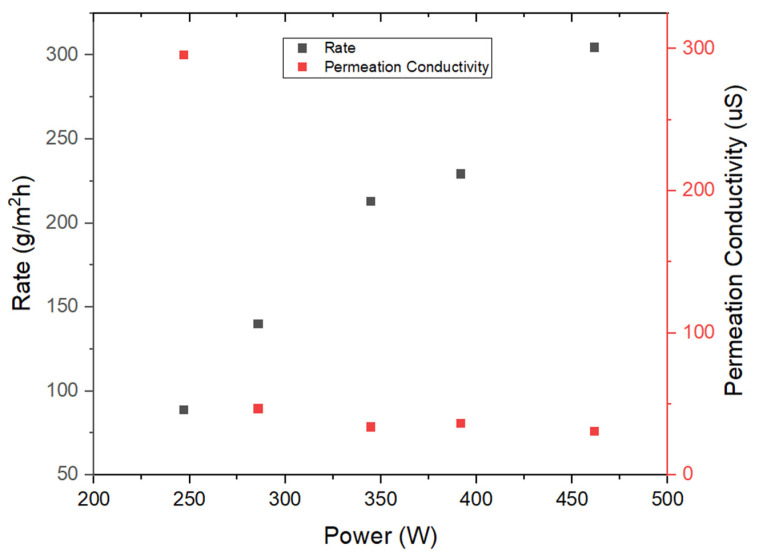
Initial lab testing results from five-layered MD unit (stainless steel thermal conducting and condensation sheets pretreated with Goop) as a function of heating pad power.

**Figure 5:**
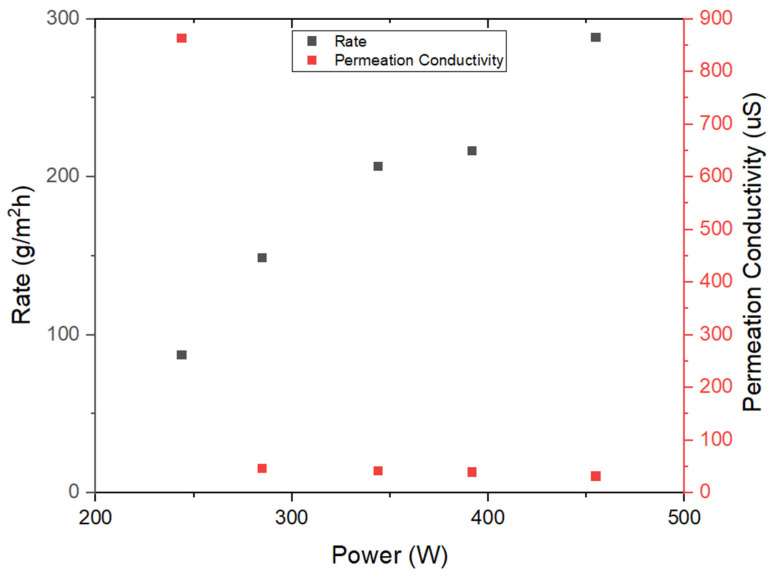
Continued lab testing results from five-layered MD unit (stainless steel thermal conducting and condensation sheets pretreated with Goop) as a function of heating pad power.

Preliminary investigations were initiated through the implementation of two 120V heating pads arranged in a serial configuration, subsequently connected to a variable transformer. The outcomes of these initial evaluations are illustrated in Figure 4 and Figure 5, corresponding to the results obtained during the first two days of experimentation. The synthetic seawater utilized in the inhouse tests as well as field tests was measured to have a conductivity between 65–70 milli-siemens (mS).

**Figure 6:**
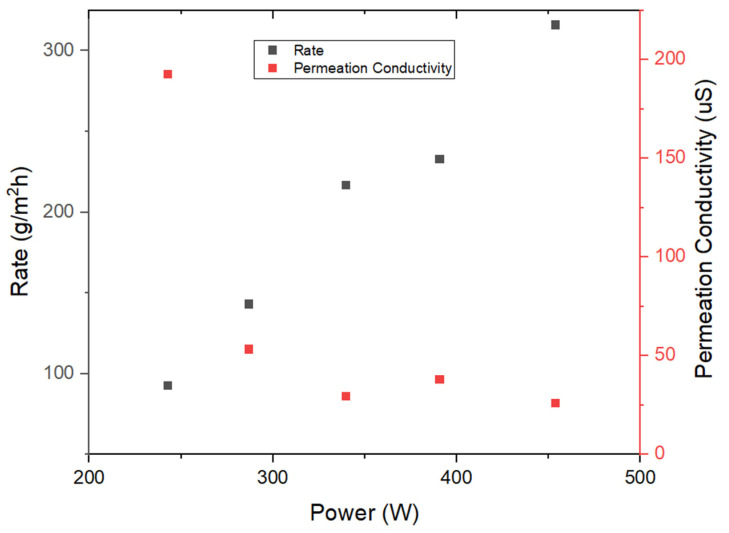
Continued lab testing results after fixing prominent engineering issues from a five-layered MD unit (stainless steel thermal conducting and condensation sheets pretreated with Goop gel) as a function of heating pad power.

Analysis of these outcomes permits the conclusion that the five-layer system demonstrates the capacity to generate pure water characterized by low conductivity levels (below 45 μS), coupled with commendable rates of water production. It is worth noting that the initial data point recorded exhibited a relatively elevated conductivity measurement, warranting an in-depth inquiry. After an extensive series of tests and investigative procedures, it was discerned that potential microleaks might originate from the feed channel. Nonetheless, the predominant source contributing to the observed conductivity levels was attributed to the gradual release of ions from the material components. Following the resolution of engineering challenges and the rectification of any structural integrity issues within the device, the results displayed in Figure 6 were obtained.

With the experimental data, it was concluded that the five-layer apparatus produces ideal fresh water that can be utilized for further experiments such as hydrogen electrolysis. With acceptable data produced, the systems were moved from lab to outdoors for field testing.

### 3.2. Field Testing Results

#### 3.2.1. Field Design 1 (Table 1)

Following the assessment of the lab apparatus, the experimental device was subsequently transitioned to outdoor field-testing setup. This outdoor counterpart retained the fundamental design principles of the lab model but was with different size measurements, now encompassing a device measuring 20 by 24 inches. The initial design configuration, elaborated upon previously and depicted in Figure 1, served as the foundational reference point.

The device was positioned in an outdoor location, characterized by ample sunlight, with the commencement of the tests each day initiated promptly at 9:00 AM. Subsequently, the system was retrieved for measurements at 4:00 PM, marking the end of each testing session. Continuous temperature monitoring was conducted throughout the day, and the cooling water was replaced throughout the day to ensure the effective management of the cooling system and to mitigate the risk of overheating.

**Table 1:** Results of Design 1 field test.

Test Number	Start Time	End Time	Net Permeation Weight (G)	Permeation Conductivity (μS)
1	9:00 AM	4:00 PM	2146.1	117.3
2	9:00 AM	4:00 PM	2392.0	135.4

This data reveals that the system consistently yielded a daily water production ranging between 2100 and 2400 g. However, it is noteworthy that the conductivity measurements exhibited a slight increase compared to the lab testing. It is imperative to acknowledge that the daily water production exhibits some degree of variation, attributable to the fluctuating weather conditions and outdoor temperatures experienced on different days. The conductivity measurements, although higher than lab results, indicate that fresh water is produced by the apparatus and that the water produced could be utilized for further experiments. These initial findings, although promising, underscore the potential for further enhancements in the design of the field test unit, with the objective of reducing energy consumption and augmenting overall system performance.

#### 3.2.2. Field Design 2 (Table 2)

The second field design was constructed utilizing the same conditioning methods and size measurements as design one. This apparatus includes a reservoir that collects the returning cooling water as well as the concentrated feed water. The feeding water was taken from this container, and the overflow of the container returns to the cooling tank. This design change increases the temperature of the feeding water, while simultaneously increasing the productivity of the device. This system still utilizes multiple peristaltic pumps to flow the water through the system requiring the same amount of energy consumption as design one. 

The testing of Design 2 was run similarly to the testing of Design 1. The device was positioned in an outdoor location, characterized by ample sunlight, with the commencement of the tests each day initiated promptly at 9:00 AM. Subsequently, the system was retrieved for measurements at 4:00 PM, marking the end of each testing session. Continuous temperature monitoring was conducted throughout the day, and the cooling water was replaced throughout the day to ensure the effective management of the cooling system and mitigate the risk of overheating.

**Table 2:** Results of Design 2 field test.

Test Number	Start Time	End Time	Net Permeation Weight (g)	Permeation Conductivity (µS)
1	9:00 AM	4:00 PM	2213.0	93.6
2	9:00 AM	4:00 PM	3234.3	132.1
3	9:00 AM	4:00 PM	3654.2	126.2
4	9:00 AM	4:00 PM	4090.8	130.9

The conducted experiments highlight the substantial enhancements achieved through the modifications introduced in the transition from the initial design to the current configuration. Notably, it is evident that there is a degree of variability present in the quantity of fresh water produced during each test. While the observed water production values have consistently exhibited an upward trajectory and reached relatively high levels, the significant variability in mass is subject for further investigation. It is conceivable that this variability is linked to the prevailing outdoor weather conditions and fluctuating temperatures, a topic to be discussed in greater detail in subsequent discussions.

Furthermore, it is imperative to acknowledge that the conductivity of the produced water shows a direct correlation with the volume of water generated, an aspect that is less than ideal. However, the observed conductivity levels remain within the established thresholds for classifying the produced water as freshwater, undergoing an increase with the increment in mass.

#### 3.2.3. Field Design 3 (Table 3)

The development process of the third field design closely paralleled the conditioning and size measurements applied in the construction of its predecessors. Like the Design 2, thermal management principles remained consistent. However, in this design, a noteworthy modification was introduced by elevating the position of the reservoir to facilitate the self-flow of feed water due to potential energy. This innovative self-flow design, which allows for the elimination of one of the peristaltic pumps, has a two-fold benefit: it reduces energy consumption and introduces a valve for the precise control of the feed water flow.

The tests for this system were conducted similarly to both Design 1 and 2.

**Table 3:** Results of Design 3 field test.

Test Number	Start Time	End Time	Net Permeation Weight (g)	Permeation Conductivity (μS)
1	9:00 AM	4:00 PM	2910.8	142.1
2	9:00 AM	4:00 PM	2401.6	131.9
3	9:00 AM	4:00 PM	2779.8	124.4
4	9:00 AM	4:00 PM	2917.6	119.6
5	9:00 AM	4:00 PM	2761.7	124.1
6	9:00 AM	4:00 PM	2693.8	131.4

Across all conducted tests, the net weight of freshwater production exhibited a good degree of uniformity, paralleled by closely aligned conductivity measurements. The system generated a consistent volume of freshwater daily, even with a reduced number of pumps in operation. This underscores that the third design has increased efficiency requiring fewer pumps to achieve successful outcomes, consequently implying a reduced energy demand from the solar panel. The power required for the pump is about 10% of the total energy generated by the solar panel. The solar panel having excess energy could be utilized for other instrumentation or other sources requiring electricity while simultaneously running this field design for freshwater creation.

### 3.3. Weather and Temperature Considerations

The weather and temperature throughout the day have a high impact on the results of the field test designs. Table 4 highlights the effects of temperature and outdoor weather on the production of freshwater.

Throughout the testing phases of each design iteration, records of temperature measurements and prevailing weather conditions were documented to assess their potential influence on freshwater production. The system is intricately dependent on the solar panel not only for powering the peristaltic pumps but also for excess heat from the solar panel to induce the required partial pressure differences. The functionality of the PV-MD system is contingent on the supplementary heat supplied by the solar panel. This process is highly dependent on the weather and solar conditions at the time of the experiment. If there was a lack of solar rays or a poor weather condition, the system would not be able to produce water consistently.

An analysis of the temperature readings strongly suggests that temperature exerts a discernible impact on the daily water production. Higher temperatures are associated with increased water production while lower temperatures result in diminished water yield. The water production values exhibit variations corresponding to differing temperature regimes. In contrast, it does not appear that conductivity is substantially affected by temperature or prevailing weather conditions, as conductivity values display variations irrespective of these factors.

### 3.4. Summary of Experimental Results

This investigation examines the impact of ambient temperature on freshwater production, revealing a positive correlation with higher temperatures enhancing the yield of low-conductivity water. The utilization of pre-heated cooling water in the membrane distillation (MD) system is identified as a strategy to improve production efficiency. Comparative analysis indicates superior performance of Design 3 over Design 2, with both configurations exhibiting enhanced efficacy relative to Design 1, signifying incremental improvements with each modification to the apparatus. The MD system’s power consumption is determined to be only 10% of the total solar panel output, thereby allowing for the allocation of the remaining 90% of power to other applications or devices. The synthetic salt water utilized exhibits a conductivity of 65–70 mS, and post-MD processing, the resulting fresh water demonstrates a significant reduction in conductivity to an average of 125 µS. This substantial decrease in ions attests to the efficacy of the designed and tested membrane distillation apparatus in selectively allowing the passage of fresh water while impeding unwanted ions through its hydrophobic membrane. Water production data, detailed in Table 4, indicates that, with a 20″ × 24″ PV-MD panel, freshwater production exceeds 3 gallons/m^2^/day at 32 °C during summer. Design 3 consistently achieves an average production of 2.2 gallons/m^2^/day at an ambient temperature of 24 °C. The findings underscore the temperature-dependent nature of freshwater production, with higher temperatures yielding increased output from the system apparatus.

## 4. Discussion

This present field study investigated the performance of three MD designs (Design 1, Design 2, and Design 3) integrated with a photovoltaic (PV) panel under real operational conditions. The results confirm that several factors significantly impact the freshwater production rate of the MD systems.

**Impact of Ambient Temperature:** A clear correlation was observed between ambient temperature and freshwater production. Higher ambient temperatures resulted in increased freshwater production, likely due to enhanced evaporation rates within the MD unit. To capitalize on this effect, pre-heating the feedwater using the cooling water from the system proved to be an effective strategy.

**Performance Comparison:** Among the tested designs, Design 3 demonstrated the highest freshwater production rate, followed by Design 2 and then Design 1. This suggests that the specific design features of Design 3 can play a crucial role in optimizing performance. Further research is needed to identify the specific design elements contributing to this improved efficiency.

**Conductivity Variation:** The initial data point in each test consistently exhibited a higher conductivity reading compared to subsequent measurements. This phenomenon could be attributed to the evaporation of residual saline water within the channels after testing. This evaporation process leads to salt crystal formation, which can potentially penetrate the membrane during drying. Upon reintroduction of water for the next test, these crystals can be dissolved, causing an initial increase in the conductivity. This high initial data point has been recognized and accounted for in the analysis.

**Energy Consumption:** The power consumption of the MD system was found to be minimal, representing only around 10% of the total power generated by the PV panel. This low energy footprint underscores the potential of the demonstrated PV/MD system for sustainable desalination applications.

**Freshwater Production:** The desalination process successfully reduced the conductivity of the saltwater feed (65–70 mS) to an average of 125 µS in the produced freshwater. Table 4 summarizes the freshwater production rates in grams/day. Notably, under favorable ambient temperatures (32 °C), the PV-MD panel achieved a production rate exceeding 3 gallons/m²/day. Even at a more moderate average temperature (24 °C), Design 3 delivered an average freshwater production rate of 2.2 gallons/m²/day. These results demonstrate the practical usage of this integrated solar-powered desalination system for providing clean water in various environmental conditions.

## 5. Conclusions

Our investigation into the construction and field testing of simulated PV-MD devices underscores the capability of our systems to yield freshwater at a low cost. The system designs in this investigation could prove to be quite beneficial in the creation of drinkable water on boats or in the off-grid environments, where the water sources include contaminated water, brackish water, and seawater. The field-tested devices underwent a multitude of assessments under varying temperature and weather conditions, with the primary goal of ascertaining a cost-effective design that can efficiently produce freshwater. The developed design exhibits immense promise by harnessing surplus heat generated by the solar panel for freshwater production while simultaneously utilizing the generated power to drive a peristaltic pump. This energy management strategy ensures that the solar panel’s capacity is not entirely exhausted, permitting its concurrent deployment for other purposes in tandem with the operation of the PV-MD system. Our tests manifest a clear temperature-dependent relationship, revealing that higher temperatures yield increased freshwater production. Comparing the results from different PV/MD system designs, it is found that Design 3 surpasses the performance of both Design 1 and Design 2. In high ambient temperatures, the third design achieves freshwater production exceeding 3 gallons/m²/day. Furthermore, the power consumption required to operate the MD system in the third design represents only a fraction of the power generated by the solar panel. This dual accomplishment underscores the efficiency of the design and its potential for sustainable and cost-effective freshwater production.

## Figures and Tables

**Table 4:** Temperature and weather conditions of runs.

Design	Weather	Highest Temp. (°C)	Production (g/day)	Average Production (g/m^2^/day)	Conductivity (μS)
1	Sunny/Partly Cloudy	29.4	2146.1	1430.7	117.3
1	Sunny	28.3	2392	1594.7	135.4
2	Sunny	23.9	2213	1475.3	93.6
2	Partly Cloudy/Sunny	32.2	3234.3	2156.2	132.1
2	Sunny	32.8	3654.2	2436.1	126.2
2	Sunny	34.4	4090.8	2727.2	130.9
3	Sunny/ Partly Cloudy	25	2910.8	1940.5	142.1
3	Sunny	22.8	2401.6	1601.1	131.9
3	Sunny/Partly Cloudy	23.3	2779.8	1853.2	124.4
3	Sunny	22.8	2917.6	1945.1	119.6
3	Sunny	25.6	2761.7	1841.1	124.1
3	Sunny	27.2	2693.8	1795.9	131.4

## Data Availability

For data supporting the reported results email corresponding author Dufei Fang.
